# Delayed gastric emptying in nondiabetic patients with end-stage kidney disease

**DOI:** 10.1080/0886022X.2022.2030754

**Published:** 2022-02-21

**Authors:** Cuiyu Wang, Chao Chen, Jin Wang, Xiaohua Guo, Yuechan. C. Deng, Li Liu, Chunmei Zhao

**Affiliations:** a Department of Nephrology, Shenzhen Hospital, Southern Medical University, Shenzhen, China; b Department of Anaesthesiology, University of Hong Kong Shenzhen Hospital, Shenzhen, China; c Department of Epidemiology, School of Public Health, Sun Yat-sen University, Guangzhou, China; d Department of Radiology, University of Hong Kong Shenzhen Hospital, Shenzhen, China

**Keywords:** Gastric emptying, end-stage kidney disease, hemodialysis, antral area, ultrasound

## Abstract

**Objective:**

This study aimed to assess the gastric emptying capacity in nondiabetic patients with end-stage kidney disease (ESKD) by ultrasound.

**Methods:**

Consecutive hemodialysis patients with ESKD (*n* = 37) and healthy controls (*n* = 37) were enrolled. All ESKD patients underwent ultrasound examinations on the day of hemodialysis (dialysis day) and the day after hemodialysis (nondialysis day). Standard ultrasound examinations were performed after overnight fasting, immediately after a light meal, and at 6 h after a meal. The antral cross-sectional area and gastric emptying according to the Perlas grading system were evaluated.

**Results:**

Compared with the controls, patients with ESKD, on both dialysis and non-dialysis days, had significantly larger antral areas when examined in the supine position (*p* = 0.002 and *p* = 0.003, respectively), but not in the right lateral decubitus position (*p* = 0.452 and *p* = 0.512, respectively). In the supine position, the antral area of ESKD patients before dialysis (8 a.m. on the dialysis day) was larger than that at the same time on the nondialysis day (*p* = 0.028). The controls had a Perlas grade of either 0 or 1 at 6 h after a meal, whereas five patients (13.5%) and 11 patients (29.7%) in the ESKD group had Perlas grade 2 on the dialysis and non-dialysis days, respectively. Among patients with or without delayed gastric emptying, no differences were detected in the dialysis duration or levels of biochemical markers, except blood urea nitrogen (*p* = 0.038) and serum creatinine (*p* = 0.003).

**Conclusion:**

Nondiabetic patients with ESKD had significantly delayed gastric emptying. Hemodialysis might improve gastric emptying and reduce gastric emptying delay.

## Introduction

Patients with end-stage kidney disease (ESKD) often have a variety of complications. Previous studies have shown delayed gastric emptying in up to one-third of patients with ESKD, whether treated with dialysis or conservative therapy [1,2]. Several studies have indicated that ESKD patients have significantly delayed gastric emptying compared with healthy counterparts [3–5]. Experiments in rats also showed that chronic renal failure leads to impaired gastrointestinal motility, altered interdigestive myoelectric activity and a lower gastric emptying rate as compared with controls [6].

In previous studies, gastric emptying was monitored mostly on the day of dialysis and sometimes on a nondialysis day; however, no studies have focused on monitoring gastric emptying on both dialysis and nondialysis days in ESKD patients. Significant changes in hemodynamics and electrolyte acid-base changes occur during the process of hemodialysis, and thus, the effect of dialysis on gastric emptying should be evaluated. Moreover, gastric emptying also should be monitored on the day after dialysis, when patients have a more stable internal environment, which can better reflect the general state of ESKD patients.

Gastric emptying abnormalities have been found in patients with diabetes mellitus [7]. However, most studies on gastric emptying in patients with ESKD did not distinguish between diabetic and non-diabetic patients. Several other factors, such as scleroderma, amyloidosis, gastrointestinal surgery and gastric ulcer disease, as well as drugs including anticholinergics and dopaminergics, may impact gastric emptying [8,9]. Therefore, consideration should be given to excluding these factors in studies of gastric emptying.

At present, several methods are available for evaluating gastric volume and gastric content in clinical practice, such as X-ray imaging, magnetic resonance imaging and radionucleotide imaging. All these methods have disadvantages, including high cost, radiation exposure, and inconvenience. In recent years, a number of studies have confirmed that the cross-sectional area (CSA) of the gastric antrum is closely associated with the volume of fluid in the stomach, and a variety of linear mathematical models have been established to predict the content of the stomach [3,10]. Manini et al. applied three-dimensional ultrasound for the measurement of gastric volume [11]. Accordingly, ultrasound is expected to become a widely used method for measuring gastric volume.

The aims of this study were to examine the gastric emptying capacity in nondiabetic patients with ESKD on both dialysis and non-dialysis days in comparison with that in healthy individuals, and to determine the relationship between gastric emptying and biochemical parameters in these patients.

## Methods

### Study participants

In this study, consecutive patients with chronic kidney disease undergoing hemodialysis at the Shenzhen Hospital, Southern Medical University from January 2020 to June 2020 were screened. The inclusion criteria included regular hemodialysis treatment for >3 months, age >8 years, body weight 45‒80 kg, height >150 cm, body mass index (BMI) <28 kg/m^2^ and ASA physical status I‒III (Class I, a healthy patient, no physiologic, physical, or psychologic abnormalities; Class II, a patient with mild systemic disease without limitation of daily activities; and Class III, a patient with severe systemic disease that limits activity but is not incapacitating). Patients were excluded if they had: diabetes; scleroderma; amyloidosis; a history of upper gastrointestinal bleeding; a history of esophageal, gastric or upper abdominal surgery; chronic gastritis or gastroesophageal reflux disease; or had taken proton pump inhibitors or gastrointestinal promotility agents within the previous two weeks. Age- and sex-matched healthy individuals without gastrointestinal diseases were enrolled as healthy controls. Control individuals were not enrolled if they had a history of active gastrointestinal ulcer, chronic gastritis and gastroesophageal reflux disease, or gastrointestinal/abdominal surgery; had presented with stomach pain, abdominal distention or diarrhea within three months prior to the study; or had severe infection within one month before the study. The study protocol was approved by our local ethics committee. Written informed consent was obtained from all participants.

The reported prevalence of delayed gastric emptying in patients with ESKD is 34%, and this rate is 3.5%‒5% in patients with ESKD after an 8-h fast [12,13]. Accordingly, the sample size calculation performed prior to the study revealed that at least 30 patients would be required in each group, in order to detect a two-sided difference (*p* < 0.05) between the two groups with a power of 80%. The demographic data of the participants as well as the causes of ESKD were collected.

### Ultrasound examinations

Each patient underwent ultrasound examinations on the day of hemodialysis and the day after hemodialysis (non-dialysis day). On the day of hemodialysis, an ultrasound examination was first performed at 8 a.m. after an overnight fast. Then, patients were provided with a light meal, which included a standardized portion of noodles, bread or rice, and clear fluid, which was eaten over 15‒30 min. Immediately after the meal, patients underwent a second ultrasound examination at about 8:30 a.m. The third ultrasound session was performed at 14:30, and the patients were allowed to drink clear water 2 h before this examination, but not allowed to eat. Routine hemodialysis treatment was performed between the second and third ultrasound examinations. On the nondialysis day, patients underwent the same imaging procedure except they did not receive hemodialysis treatment.

A curved, low-frequency 2–5-MHz probe was used for measurements with the GE Healthcare Venue 50 ultrasound system (GE Healthcare, Wauwatosa, WI, USA). Standard ultrasound examinations were performed by the same ultrasound technician. Estimations of gastric contents were carried out with patients in both the supine and right lateral decubitus positions. The anterio-posterior (AP) and cranio-caudal (CC) diameters were measured, and the CSA estimated using the formula: CSA=(AP × CC × π)/4 [2].

Gastric contents were qualitatively evaluated with the three-level grading system described by Perlas et al [14], where: Grade 0 is defined by an empty antrum in both the supine and the right lateral positions; Grade 1 is defined by fluid detection only in the right lateral decubitus position; and grade 2 is defined by fluid or food seen in the both supine and right lateral decubitus positions. Delayed gastric emptying was defined as a Perlas grade 2 either after an overnight fast or a 6-h daytime fast.

### Statistical analysis

All statistical analyses were performed using SPSS 23.0. Quantitative data are presented as the mean ± standard deviation (SD) or median and quartiles (upper and lower quartiles). Comparisons of continuous variables between the groups were performed using the independent *t*-test or one-way analysis of variance (ANOVA) for normally distributed data and the nonparametric Mann–Whitney U-test for data that were not normally distributed. Categorical data are expressed as number and percentage and were compared using chi-squared test or Fisher’s exact test. The antral CSA was analyzed and compared using repeat-measures ANOVA. *p* < 0.05 indicated statistical significance.

## Results

A total of 55 ESKD patients were eligible for inclusion, and 18 were excluded due to administration of proton pump inhibitors (*n* = 4), gastrointestinal promotility agents (*n* = 3) or both (*n* = 3); having recurrent diarrhea (*n* = 2), gastric ulcer (*n* = 3), or gastroesophageal reflux disease (*n* = 1); or refusal to participate (*n* = 2). Finally, 37 ESKD patients and 37 controls were enrolled. Among the 37 ESKD patients included, 34 used an arteriovenous fistula and three used a long-term hemodialysis catheter for hemodialysis vascular access. All patients underwent hemodialysis three times a week. The demographic characteristics of the patients and the causes of renal failure are presented in Tables 1 and 2.

**Table 1.** Characteristics of participants.

	Controls (*n* = 37)	Patients with ESRD (*n* = 37)	*P* value
Age, years	44.7 ± 11.9	44.8 ± 11.8	0.953
Male, n (%)	22 (59.5)	22 (59.5)	1.000
Weight, kg	62.1 ± 7.5	60.4 ± 10.4	0.433
Height, cm	1.65 ± 0.09	1.65 ± 0.06	0.884
BMI, kg/m^2^	22.8 ± 1.8	22.1 ± 3.1	0.263
Cause of renal failure (*n*)			
Chronic nephritis	‒	8	
IgA nephropathy	‒	6	
Hypertension	‒	2	
Polycystic kidney	‒	2	
Systemic lupus	‒	1	
Kidney stone	‒	1	
Obstruction	‒	1	
Tuberculosis of kidney	‒	1	
Unknown	‒	15	
Blood urea nitrogen, mmol/l	4.2 ± 0.8	22.8 ± 4.9	<0.001
Serum creatinine, mmol/l	63.9 ± 12.9	1015.4 ± 242.8	<0.001
K, mmo/l	4.1 ± 0.3	4.6 ± 0.6	<0.001
Na, mmo/l	141.5 ± 1.9	140.0 ± 3.3	0.006
Ca, mmo/l	2.3 ± 0.1	2.2 ± 0.2	0.083
PO4, mmol/l	1.1 ± 0.1	2.2 ± 1.5	<0.001
Hemoglobin, g/l	139.8 ± 10.1	109.3 ± 17.2	<0.001
Albumin, g/l	45.2 ± 2.85	41.1 ± 3.7	<0.001

**Table 2.** Estimated antral CSA and Perlas grade after a 6-h fast in participants.

	Patients with ESKD (*n* = 30)		Controls (*n* = 30)	*P* values*		
	Dialysis day	Non-dialysis day		Group effect	Session effect	Interaction effect
Antral area; supine						
Fasted	435.2 ± 205.5	354.7 ± 123.7	310.5 ± 81.9	0.002^a^	<0.001^a^	0.236^a^
After meal	1084.0 ± 333.4	1101.8 ± 362.0	887.4 ± 388.6	0.003^b^	<0.001^b^	0.065^b^
6 h after meal	400.9 ± 150.4	408.2 ± 129.7	312.5 ± 108.3	0.660^c^	<0.001^c^	0.231^c^
Antral area; RLD						
Fasted	585.1 ± 189.8	545.6 ± 175.6	494.8 ± 245.8	0.452^a^	<0.001^a^	0.141^a^
After meal	1528.6 ± 408.8	1274.3 ± 348.7	1606.2 ± 651.3	0.512^b^	<0.001^b^	<0.001^b^
6 h after meal	563.6 ± 218.8	614.6 ± 286.5	446.1 ± 213.1	0.087^c^	<0.001^c^	0.002^c^
Perlas grade 6 h after meal, n (%)						
0	26 (70.3)	14 (37.8)	28 (75.7)	0.065^a^		
1	6 (16.2)	12 (32.4)	9 (24.3)	<0.001^b^		
2	5 (13.5)	11 (29.7)	0	0.024^c^		

CSA: cross-sectional area; ESRD: end-stage renal disease; HD: hemodialysis; RLD: right lateral position.

*Comparison was made between ^a^controls versus patients on dialysis day, ^b^controls versus patients on non-dialysis day and ^c^dialysis versus nondialysis days.

**Figure 1.**
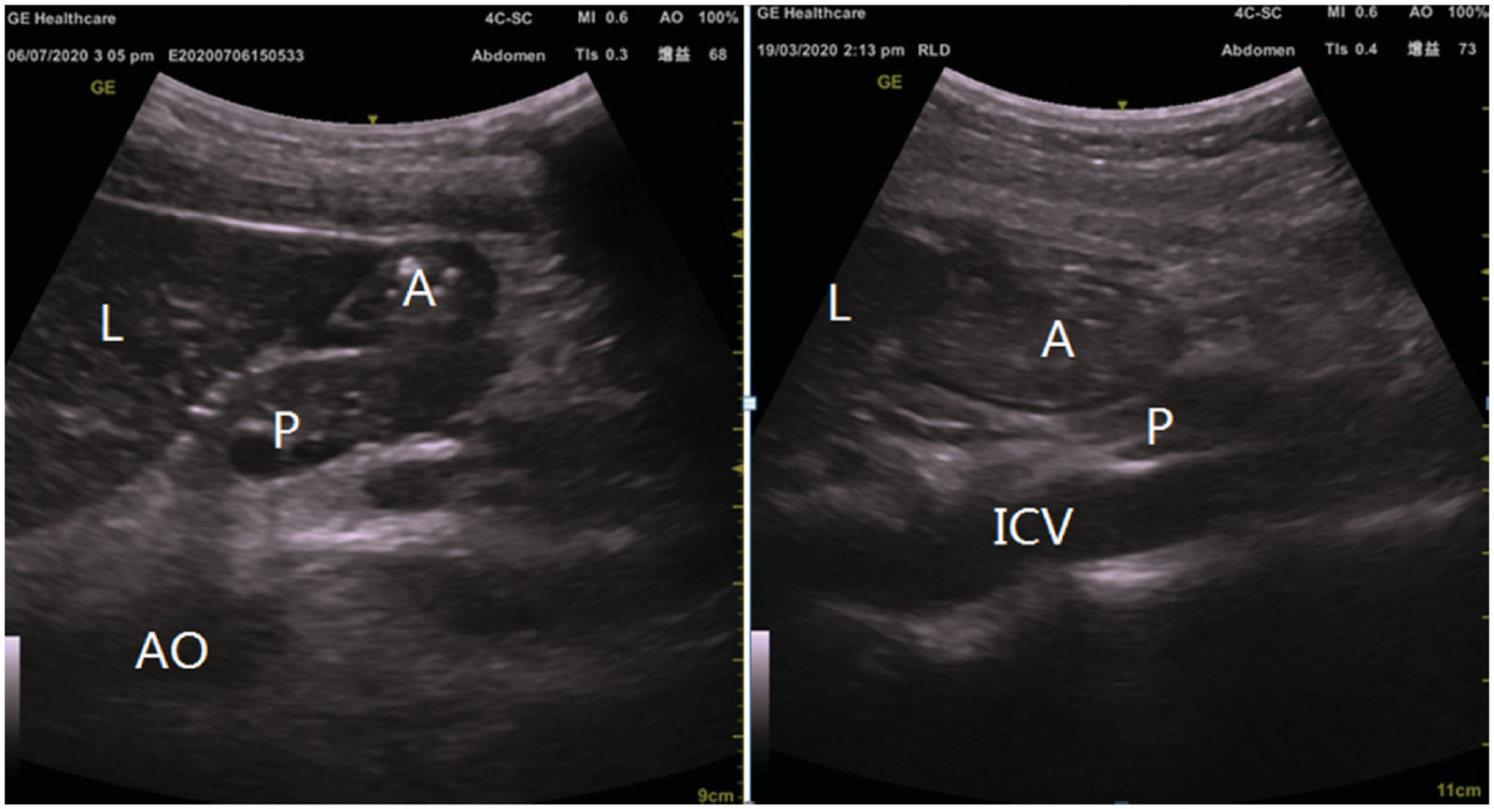
**Ultrasound images**. Representative ultrasound images showing a gastric antrum in the supine position (A) after overnight fasting and (B) at 5 min after a meal. A: antrum; L: liver; P: pancreas; IVC: inferior vena cava.

**Figure 2.**
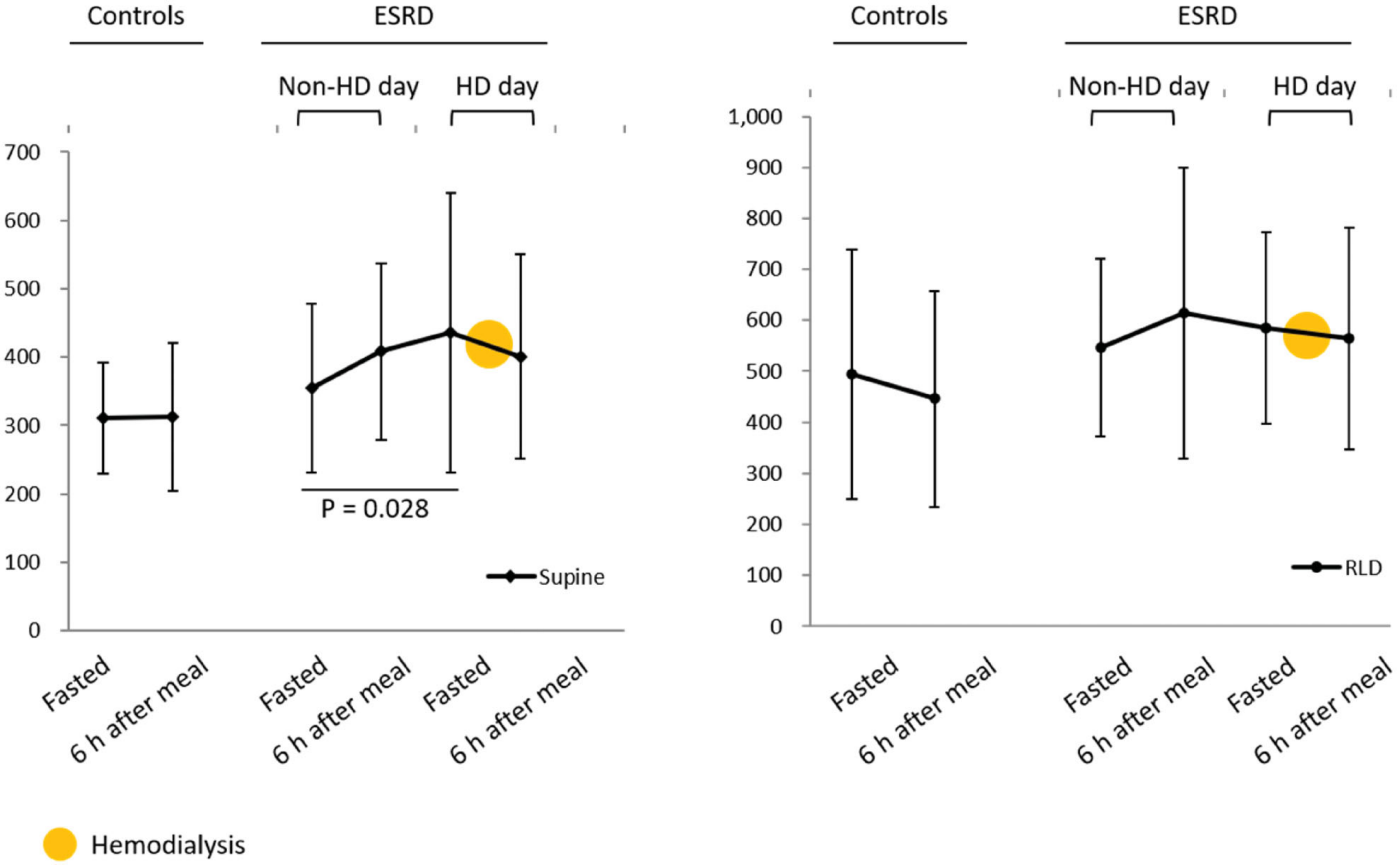
Estimated antral CSA measured in the supine (A) and right lateral positions (B) in patients with ESKD and control participants. No significant differences in antral area were found between nonhemodialysis day and hemodialysis day or between the fasted condition and 6 h after a meal. CSA: cross-sectional area; ESKD: end-stage kidney disease; RLD: right lateral position; HD: hemodialysis.

The antral area was measured after fasting, immediately after a meal, and at 6 h after a meal on both dialysis and non-dialysis days (Figure 1). In all groups, the antral area was significantly increased after a meal as compared with the fasted state, but returned to baseline values by 6 h after the meal (*p* < 0.001, Table 3). Compared with the controls, patients with ESKD, on both dialysis and nondialysis days, had a significantly larger antral area when examined in the supine position (*p* = 0.002 and *p* = 0.003, respectively), but not the right lateral decubitus position (*p* = 0.452 and *p* = 0.512, respectively). However, no significant difference in antral area was observed in ESKD patients between dialysis and non-dialysis days, in either the supine (*p* = 0.660) or right lateral (*p* = 0.087) position. Curves for the estimated antral area after overnight fasting and at 6 h after a meal were plotted (Figure 2). In the supine position, the antral area of ESKD patients on the day of dialysis (8 a.m.) was larger than that on the non-dialysis day (8 a.m., *p* = 0.028). No other significant differences in antral area were found between dialysis and nondialysis days, or between the conditions of fasted and 6-h postmeal in ESKD patients.

**Table 3.** Comparison of clinical characteristics in ESKD patients between those with normal or delayed gastric emptying at 6 h after a meal on non-dialysis day.

	Normal emptying (*n* = 26)	Delayed emptying (*n* = 11)	*P* value
Age, years	46.7 ± 11.7	40.2 ± 11.2	0.128
BMI, kg/m^2^	21.6 ± 2.8	23.3 ± 3.4	0.133
Hemodialysis duration, months	11 (5, 48.5)	12 (7, 54)	0.748
Blood urea nitrogen, mmol/l	21.7 ± 4.6	25.3 ± 5.0	0.038
Serum creatinine, mmol/l	941.9 ± 173.2	1189.1 ± 300.0	0.003
K, mmo/l	4.7 ± 0.6	4.5 ± 0.5	0.459
Na, mmo/l	140.0 ± 3.5	140.0 ± 2.6	0.972
Ca, mmo/l	2.2 ± 0.2	2.1 ± 0.2	0.148
PO_4_, mmol/l	1.86 (1.55, 2.18)	1.99 (1.74, 2.51)	0.408
Parathyroid hormone, pg/ml	232.6 (151.5, 448.0)	238.9 (30, 394.5)	0.915
Hemoglobin, g/l	108.7 ± 16.8	110.7 ± 18.8	0.752
Albumin, g/l	40.8 ± 3.2	41.6 ± 4.7	0.525
Kt/V	1.47 ± 0.15	1.36 ± 0.28	0.103
Urea reduction ratio (%)	71.1 ± 5.8	68.8 ± 6.5	0.301

Kt/V, urea clearance × dialysis duration (min)/urea V_D_.

Normal gastric emptying: Perlas grade 0 and 1; delayed gastric emptying: Perlas grade 2.

Data were presented as the mean ± SD or median (upper and lower quartiles).

With regard to gastric contents, all participants showed Perlas grade 0 after overnight fasting and grade 2 after the meal. In the control group, the Perlas grade was either 0 (*n* = 28, 75.7%) or 1 (*n* = 9, 24.3%) at 6 h after a meal. However, five patients (13.5%) and 11 patients (29.7%) in the ESKD group had grade 2 on the dialysis and nondialysis days, respectively (Table 3).

Between patients with or without delayed gastric emptying, no differences were found in the duration of dialysis or levels of biochemical markers, with the exceptions of blood urea nitrogen (*p* = 0.038) and serum creatinine (*p* = 0.003) (Table 3).

## Discussion

This is the first study to evaluate gastric emptying in nondiabetic patients with ESKD. In this study, all participants had an empty stomach after an overnight fast. None of the controls had delayed gastric emptying; in contrast, 11 of 37 patients with ESKD (29.7%) had delayed gastric emptying on the non-dialysis day, and five of 37 (13.5%) patients still had significant residual gastric contents after 4 h after hemodialysis. We also found that patients with ESKD had a larger antral CSA when measured in the supine position compared with the control participants, but the difference was not statistically significant when measurements were taken in the right lateral position. The results of the study indicated that non-diabetic patients with ESKD had significantly delayed gastric emptying, and hemodialysis might improve gastric emptying and reduce gastric emptying delay.

The mechanism by which chronic kidney disease induces gastric hypomotility is unclear and probably multifactorial. Patients with ESKD are prone to clinical disorders, such as heavy water load, electrolyte disturbance, and acidosis. In addition, uremic patients often also have arteriosclerosis and anemia, both of which affect the local blood supply of the gastric mucosa, leading to defects in gastric barrier function and abnormal gastrointestinal motility. Furgala et al. demonstrated gastric motility disorders in uremic patients by detecting gastric myoelectric activity [15]. Therefore, gastric residues with pathogenic microorganisms and toxins that cannot removed in a timely manner may affect the function of the gastrointestinal tract. The kidney is the site of degradation and clearance of gastrointestinal peptide hormones [16]. Changes in gastrointestinal peptide hormones have been observed in patients with acute and chronic renal failure [17]. Certain gastrointestinal hormones that regulate mobility, such as cholecystokinin and gastrin, may be increased [18,19]. More importantly, cytokines and uremic toxins that affect the autonomic and enteric nervous systems may alter the myoelectrical activity of gastric smooth muscle or pacemaker cells, causing the observed changes in gastrointestinal function [15]. Moreover, the disorders of calcium and phosphorus metabolism secondary to hyperparathyroidism further disrupt the secretion of gastrointestinal hormones.

Studies have shown that hemodialysis improves gastrointestinal motility and reduces gastrointestinal symptoms in patients with ESKD [20]. A study demonstrated a reversal of gastric mucosal atrophy with regular hemodialysis in patients with chronic kidney disease [21]. Gastrointestinal symptoms, including abdominal distention, nausea, acid regurgitation and delayed gastric emptying, occur in approximately 70% of patients with renal failure [22]. After regular hemodialysis, the gastrointestinal symptoms of patients are significantly improved, with a shortened gastric emptying time. Our study showed an improvement in gastric emptying delay during hemodialysis, which might be related to the reduction of uremic toxins. Also, patients with delayed gastric emptying had significant higher blood urea nitrogen and serum creatinine levels compared with those with normal gastric emptying. Previous studies have shown that the delay of gastric emptying is associated with changes in nutritional status such as serum albumin and prealbumin [12]. In our study, the albumin levels of the patients were all in the normal range, and none of the patients had hypoalbuminemia. Also, we observed no significant differences in the duration of dialysis or levels of nutritional status markers between patients with normal or delayed gastric emptying. The specific mechanism requires further investigation.

Our results showed that in the supine position, the antral area of patients peaked after overnight fasting on the dialysis day, gradually decreased after hemodialysis treatment to a minimum on the non-dialysis day, and then increased again to form a cycle. In addition, a higher proportion of patients had delayed gastric emptying on the non-dialysis day (29.7%) than on the dialysis day (13.5%) as determined at 14:30 after hemodialysis, although the difference did not achieve statistical significance (*p* = 0.065) probably due to the relatively small sample size. These data suggest that hemodialysis might improve gastric emptying and reduce gastric emptying delay.

In this study, we found that gastric emptying in non-diabetic patients with ESKD. Our results suggest that gastric emptying was delayed in nondiabetic ESKD patients on both dialysis and nondialysis days as compared with that in the healthy controls. Our results are consistent with the majority of previous studies and show that hemodialysis might improve gastric emptying and reduce gastric emptying delay. Future research on gastric emptying in ESKD patients, including measurement of gastric emptying time, is needed with a larger sample size.
